# A clinical decision support system is associated with reduced loss to follow-up among patients receiving HIV treatment in Kenya: a cluster randomized trial

**DOI:** 10.1186/s12911-021-01718-0

**Published:** 2021-12-20

**Authors:** Tom Oluoch, Ronald Cornet, Jacques Muthusi, Abraham Katana, Davies Kimanga, Daniel Kwaro, Nicky Okeyo, Ameen Abu-Hanna, Nicolette de Keizer

**Affiliations:** 1 Division of Global HIV and TB, US Centers for Disease Control and Prevention, 1600 Clifton Road NE, GA 30329 Atlanta, USA; 2 Department of Medical Informatics, Amsterdam Public Health Research Institute, Amsterdam UMC, University of Amsterdam, Amsterdam, The Netherlands; 3 Division of Global HIV and TB, US Centers for Disease Control and Prevention, Nairobi, Kenya; 4 Kenya Medical Research Institute – CDC Collaborative Program, Kisumu, Kenya

**Keywords:** Electronic medical records, HIV, Loss to follow-up, Decision support systems, Quality of care, Low resource country

## Abstract

**Background:**

Loss to follow-up (LFTU) among HIV patients remains a major obstacle to achieving treatment goals with the risk of failure to achieve viral suppression and thereby increased HIV transmission. Although use of clinical decision support systems (CDSS) has been shown to improve adherence to HIV clinical guidance, to our knowledge, this is among the first studies conducted to show its effect on LTFU in low-resource settings.

**Methods:**

We analyzed data from a cluster randomized controlled trial in adults and children (aged ≥ 18 months) who were receiving antiretroviral therapy at 20 HIV clinics in western Kenya between Sept 1, 2012 and Jan 31, 2014. Participating clinics were randomly assigned, via block randomization. Clinics in the control arm had electronic health records (EHR) only while the intervention arm had an EHR with CDSS. The study objectives were to assess the effects of a CDSS, implemented as alerts on an EHR system, on: (1) the proportion of patients that were LTFU, (2) LTFU patients traced and successfully linked back to treatment, and (3) time from enrollment on the study to documentation of LTFU.

**Results:**

Among 5901 eligible patients receiving ART, 40.6% (n = 2396) were LTFU during the study period. CDSS was associated with lower LTFU among the patients (Adjusted Odds Ratio—aOR 0.70 (95% CI 0.65–0.77)). The proportions of patients linked back to treatment were 25.8% (95% CI 21.5–25.0) and 30.6% (95% CI 27.9–33.4)) in EHR only and EHR with CDSS sites respectively. CDSS was marginally associated with reduced time from enrollment on the study to first documentation of LTFU (adjusted Hazard Ratio—aHR *0.85 (95% CI 0.78–0.92)*).

**Conclusion:**

A CDSS can potentially improve quality of care through reduction and early detection of defaulting and LTFU among HIV patients and their re-engagement in care in a resource-limited country. Future research is needed on how CDSS can best be combined with other interventions to reduce LTFU.

*Trial registration* NCT01634802. Registered at www.clinicaltrials.gov on 12-Jul-2012. Registered prospectively.

## Background

The 2018 version of the report of the Joint United Nations Program on HIV/AIDS (UNAIDS) indicates that approximately 23.3 million (61.5%) of 37.9 million HIV-infected people globally are on life-saving antiretroviral therapy (ART). Sub-Saharan Africa (SSA) is home to 25.6 million HIV-infected persons of which 64% (n = 16.4 million) were receiving ART at the end of 2018 [1]. The UNAIDS Fast-Track goals, commonly referred to as the 95–95–95 goals, recommend that countries should have 95% of all HIV-infected persons know their HIV status, 95% of those who know their HIV positive status initiated on ART and 95% of those on treatment achieving viral suppression by 2030 in order to end the AIDS pandemic [2]. Good adherence to treatment is essential in achieving viral suppression and reduction in HIV transmission [3]. Many countries in SSA still suffer high loss to follow-up (LTFU) of patients on ART, poor adherence to treatment and low retention rates. Studies have shown that LTFU in SSA countries could be as high as 40% among the general population after 36 months of enrollment on treatment [4–6] and as high as 57% among youth [7].

While patient demographics, behavior and related factors such as limited knowledge of the need for good adherence and appointment keeping significantly contribute to LTFU [7–9], effective alerts in clinical information systems used by care providers to identify patients who miss their appointments and strong tracking systems could potentially reduce cases of LTFU. Previous studies have shown that EHRs with CDSS can effectively track clinic attendance, flag individual patients who fail to show up for their appointment [10]. Once identified, community-based social workers perform patient tracing through phone calls and home visits for defaulting patients and those that are LTFU, and provide appropriate education, counseling and support to ensure they return to the clinic to continue treatment in accordance with Kenya national HIV treatment guidelines [11]. The weak data systems used in SSA are often incapable of providing timely information on patients who have transferred to other clinics, have died or have missed their monthly appointments. Innovative means such as computerized reminders in electronic health records (EHR) have been shown to improve patient follow-up in chronic care and adherence to treatment guidelines at population and individual patient levels [12, 13].

EHR with a clinical decision support system (CDSS), implemented as alerts or reminders that are displayed on a computer screen in the clinic or printed out routinely, have been used to provide information on selected clinical and process indicators that often improve individual patient care through better adherence to guidelines. Such indicators include trends and thresholds in vital signs, treatment history, co-infections and clinic attendance/appointments [14, 15]. A CDSS often recommend appropriate action to be taken after comparing specific patient parameters to pre-determined values stored in the EHR’s internal database based on guidelines. A systematic review by van de Velde et al.showed that CDSSs could be more effective when suggestions are patient-specific compared to group-based recommendations [16]. Automated alerts and reminders with actionable recommendations are increasingly used as key tools for HIV care as the number of patients enrolled on ART increases and health facilities need efficient, accurate and reliable systems for early identification and appropriate follow-up action on those that are LTFU. There are very few studies describing the association between the use of CDSS and LTFU. To our knowledge, this is among the first papers to show the effect of an alert-based CDSS on LTFU in a low-resource setting.

## Methods

We conducted a prospective, cluster randomized controlled study in Siaya County, western Kenya to assess the effect of an EHR with CDSS compared to EHR only on timely identification of patients experiencing immunological treatment failure and appropriate action taken [17]. Data collection and follow-up period at each site was 12 months but sites had varying start dates within the study period to allow for facility readiness. At the end of 12 months, each site had achieved the allocated sample size and data collection was stopped. The study was conducted, and reported, in adherence to the CONSORT extension for cluster trials guidelines. In this paper we report on a secondary analysis of the data to assess the effect of a CDSS on LTFU of patients receiving ART at the study sites.

### Setting and patient population

Siaya county, where the study was conducted, has one of the highest HIV prevalence in Kenya. Approximately 17.8% of adults aged 15–49 were HIV-positive compared to the national prevalence of 5.6% [10]. The study sites consisted of 20 health facilities where the Kenya Medical Research Institute (KEMRI) provides data management support for routine health service delivery and research.

All patients aged two years or older were included in the study. We included all patients who were already receiving ART three months prior to implementation of the EHR at the clinic and during the data collection period but excluded those that were newly initiating treatment after the 9^th^ month of the study since the follow-up time within the study period would only be three months (inadequate time to tell if the patients were LTFU as defined in the MOH Guidelines). Participants had varied follow-up time depending on when they initiated ART at the study site.

### Lost to follow-up (LTFU) patients

The Kenyan Ministry of Health’s (MOH) HIV treatment guidelines (adapted from WHO’s HIV consolidated treatment guidelines, 2012) describes a patient that is LTFU as: *“a client who has not turned up or come back to the clinic for either a clinical visit or refills for more than 90 days (3 months) from the last scheduled visit”* [11, 18]. Before a patient is classified as LTFU, he/she is considered a *Defaulter*. According to the Kenya MOH guidelines: *“A defaulter is a client who has not turned up for either a clinical visit or refills 7 days after their scheduled appointment date* [11]. In clinics where paper-based systems are used to document patients treatment records, the daily (or in some cases weekly) appointment list is prepared by manually reviewing individual patient charts and retrieving the date of next visit. At the end of each clinic day, the staff responsible for data management (often data clerks or nurses) review the Daily Attendance Register to identify names of patients who missed their appointments and this is used to classify defaulting patients or those that are LTFU before tracing is initiated through social or community health workers. Timely tracing enables the community health worker to offer the necessary education, counseling and support to the patient and refer them back to the clinic to resume treatment.

### Randomization

Of the 20 facilities where KEMRI provides data management support, seven were excluded from the study as they did not have reliable electric power, a secure location for a computer, or permanent data clerks to help with the regular data management activities. Each health facility was considered a cluster due to the uniformity of care offered to the patients. Allocation to study arms was at facility/clinic level and all eligible patients receiving HIV treatment at participating facilities were automatically assigned to the arm of the study to which the clinic was assigned. The KEMRI data management team used block randomization to assign the eligible 13 health facilities into two groups—EHR only (n = 6) or EHR plus CDSS (n = 7), matched by the MOH level and number of patients enrolled on HIV care. Level 2 facility (Dispensary) is defined as: headed by a nurse, offers basic out-patient and some preventive services; Level 3 (Health Center), headed by a clinical officer, offers out-patient, maternal child health and limited in-patient services; Level 4 (District Hospital), headed by a physician, is a district referral facility and offers emergency, outpatient and in-patient services [19]. For each MOH level, whenever a clinic was assigned to the EHR with CDSS group through a random selection, a same-level clinic with comparable number of patients on HIV care was assigned to the EHR-only group. Each group had 1–3 levels of health facilities. Level 1 (Community clinics) were not included since they don’t offer HIV treatment services. The KEMRI data management team were not involved in data analysis and the CDC statisticians who performed the analysis were blinded to the allocation of clinics into the respective arms of the study.

### LTFU and Electronic Health Records

In clinics with EHR systems, appointment lists are automatically generated from the computerized system at the start of the clinic day. Lists of defaulters and patients that are LFTU are automatically generated at the end of each week. The 20 HIV clinics in Siaya County where KEMRI supported data management had an EHR system referred to as Comprehensive Care Centre Patient Application Database (C-PAD). The C-PAD EHR was originally developed as a standalone application using Visual Basic for Applications in 2007. It underwent several enhancements and a CDSS was integrated into the 2012 version prior to the start of this study. Following the randomization described above, the intervention group had an EHR with CDSS functionality while the CDSS was turned off (muted) in the control group. The main difference between the two systems is that the version with a CDSS identifies individuals that are LTFU and recommends appropriate action at individual level (included in the patient charts) while the version that is an EHR-only does not make any recommendations beyond generating a weekly list of all patients who missed appointments. Health workers in the sites with EHR and CDSS were trained on the appropriate action to take whenever alerts were encountered. Such action included immediate follow-up of patient or inclusion of a note in the patient chart for action during the next clinic visit.

For the two study groups (EHR only and EHR + CDSS), clinicians recorded data on the paper form (the so-called blue card) during the consultation, and the data clerk entered the data into the computer on the same day of clinic visit. For patients in the EHR + CDSS group who miss an appointment and meet the criteria for defaulter or LTFU, the system generates an alert with the patient’s last visit date, date of the missed appointment and number of days since the appointment date and whether they are considered defaulters or LTFU. This information is printed out and included in the individual patient charts with recommendation for appropriate action such as tracing defaulters or revising documentation of status (LTFU, transferred out or dead). The main effect of the intervention is to inform timely tracing of the defaulting patients or those that are LTFU. In the EHR only (usual care) group, the alerts were turned off in the instance of the EHR installed and there were no individual patient level alerts printed out nor recommendations filed in the patient charts; the clinical staff relied on weekly summary reports which list all patients who missed appointments in order to make decisions on follow-up.

KEMRI data managers routinely reviewed the data and any missing or unusual values were sent back to the clinician via the data clerks for completion, correction or confirmation.

### Outcome measures

The primary outcome measure for this study was the proportion of patients receiving HIV treatment that were LTFU at least once during the study period. Secondary outcomes measures were the proportion of LTFU patients traced and successfully linked back to treatment within the study period, and time from enrollment on the study to documentation of LTFU.

### Data management

The KEMRI data management team abstracted selected variables from the EHR. Individual patient records were de-identified and assigned study numbers that could not be traced back to the patient. Analytic datasets were created and duplicate entries deleted. Such duplicate entries may have resulted from erroneous creation of new records for patients who could not be correctly identified at the registration desk during clinic visits but were eventually correctly matched and linked to previous visits. Patients were coded as LFTU if they met the MOH’s definition. Those that were LTFU but were traced and referred back to the facility and successfully re-initiated treatment were still counted as LTFU. Approximately 15% of patients that were LTFU, were lost more than once during the study period and the proportions were comparable across the groups. We excluded from analysis, records of patients who only had one documented clinic visit during the study period to ensure that transit patients visiting the clinic for drugs refill only or had not made up their minds about permanently enrolling on care at the health facility were not mistakenly counted as LTFU.

### Statistical analysis

The sample size calculation was adapted from the method used in the main study reported in [17]. We calculated means with 95% confidence intervals and medians with inter-quartile ranges to summarize continuous variables. We used the Kruskal–Wallis test to compare distribution of medians and ANOVA to test for mean differences by outcome status. We used generalized estimating equations to analyze clustered data to determine predictors of LTFU over time and Cox proportional hazard regression to identify risk factors associated with time to documentation of first loss to follow-up. We used Kaplan–Meier survival plots and obtained hazard ratios from the clustered Cox regression to estimate the effect size of the intervention on the time-to-event outcomes and reported on the corresponding *p* values. Data were censored at the last follow-up visit. The multi-variable analysis was adjusted for the following patient-level covariates: age, sex, marital status, CD-4 category, WHO stage, and treatment regimen; and site-level variable (level of health facility). We used Stata (version 14.0) [Stata Corp, Austin, Texas] and Statistical Analysis Software (SAS® 9.4 Base SAS. Cary, NC: SAS Institute Inc., 2014) for both data management and statistical analysis.

### Missing data considerations

The data contained missing values for some of the patient-level covariates. We compared the results from a complete case analysis (CCA) and multiple imputation (MI) and selected the MI method for our analysis as it reduces bias and provides more efficient inferences since we could not tell with certainty that data were missing completely at random (MCAR). The Markov chain Monte Carlo method was used to impute missing data. In the logistic regression model, variables with a high proportion of missing data (> 30%) were dropped from the analysis.

### Ethical review

The study was reviewed in accordance with the Centers for Disease Control and Prevention (CDC) human research protection procedures and was determined to be research, but CDC investigators did not interact with human subjects or have access to identifiable data or specimens for research purposes. The Kenya Medical Research Institute’s (KEMRI) Ethical Review Committee reviewed and approved the study. All data were de-identified by the KEMRI staff participating in this study prior to analysis.

This trial is registered with ClinicalTrials.gov, number NCT01634802.

## Results

**Fig. 1:**
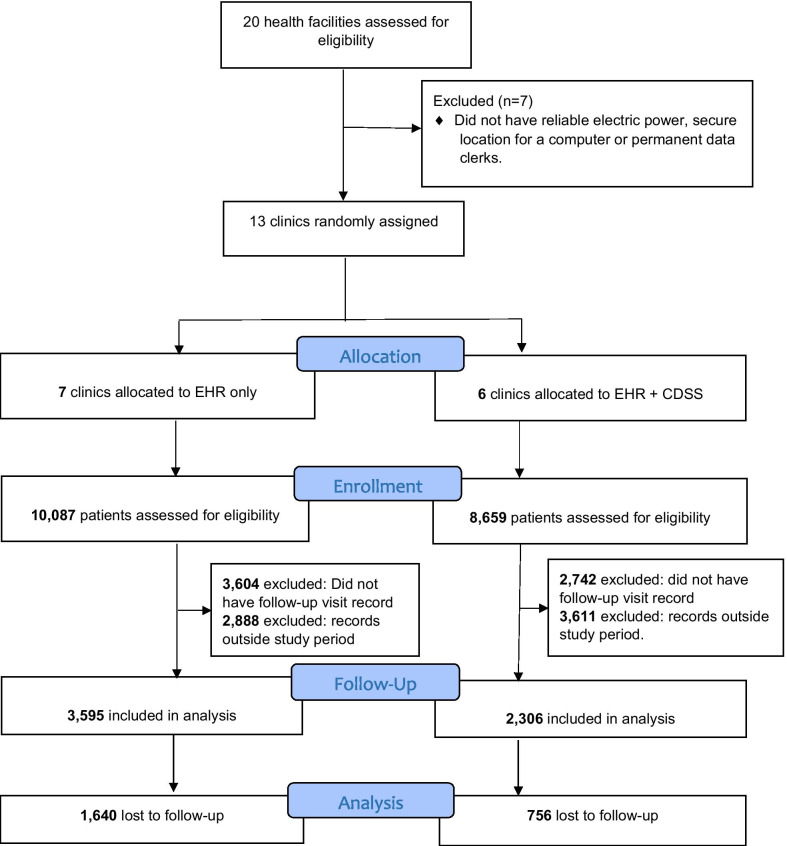
The study profile

The study was conducted between September 1st, 2012 and January 31st, 2014, during which 13 eligible clinics were randomly assigned to the control (n = 7) or intervention (n = 6) arms (Fig. 1). A total of 5901 patients who had at least one clinic visit three months prior to the installation of the CDSS and those initiating ART during the first 9 of the 12 months of the study were included. Of those included in the analysis, 3595 (60.9%) and 2306 (39.1%) were in the control and intervention arms respectively. Patients aged 30–39 years were the majority—32.5% (control) and 33.2 (intervention) while a higher number of females were registered in both arms of the study (63.5% and 61.1% in control and intervention arms respectively) compared to men. Of all the patients in the study, 52.5% were married, 42.2% had a CD4 count below 200 cells/µl and 43.1% and 2.8% were classified in WHO stages III and IV respectively (indicative of active illness). We noted statistically significant differences in weighted proportions (%) between the control and intervention arms in a few categories of some variables such as Age (50–59 years category: 7.0 (95% CI 6.1–7.8) vs. 9.1 (95% CI 7.9–10.3)) and CD4 (< 200 cells/µl category: 44.3 (95% CI 42.7–45.9) vs. 39% (37.0–41.0)) (Table 1).

**Table 1:** Baseline characteristics of CDSS art patients by site type, n = 5901

Characteristic	Control			Intervention			Total			P value
	Unweighted n	Weighted %	95% CI	Unweighted n	Weighted %	95% CI	Unweighted N	Weighted %	95% CI	
*Age group, years*										< .001
< 10 years	303	8.4	(7.5–9.3)	170	7.4	(6.3–8.4)	473	8	(7.3–8.7)	
10–19 years	128	3.6	(3.0–4.2)	82	3.6	(2.8–4.3)	210	3.6	(3.1–4.0)	
20–29 years	1065	29.6	(28.1–31.1)	638	27.7	(25.8–29.5)	1703	28.9	(27.7–30.0)	
30–39 years	1169	32.5	(31.0–34.0)	766	33.2	(31.3–35.1)	1935	32.8	(31.6–34.0)	
40–49 years	610	17	(15.7–18.2)	359	15.6	(14.1–17.0)	969	16.4	(15.5–17.4)	
50–59 years	251	7	(6.1–7.8)	210	9.1	(7.9–10.3)	461	7.8	(7.1–8.5)	
60 + years	69	1.9	(1.5–2.4)	81	3.5	(2.8–4.3)	150	2.5	(2.1–2.9)	
Total	3595	100	(–)	2306	100	(–)	5901	100	(–)	
*Sex*										0.055
Male	1311	36.5	(34.9–38.0)	898	38.9	(37.0–40.9)	2209	37.4	(36.2–38.7)	
Female	2284	63.5	(62.0–65.1)	1408	61.1	(59.1–63.0)	3692	62.6	(61.3–63.8)	
Total	3595	100	(–)	2306	100	(–)	5901	100	(–)	
*Marital status*										< .001
Missing	309	8.6	(7.7–9.5)	202	8.8	(7.6–9.9)	511	8.7	(7.9–9.4)	
Married	1934	53.8	(52.2–55.4)	1163	50.4	(48.4–52.5)	3097	52.5	(51.2–53.8)	
Divorced/separated	138	3.8	(3.2–4.5)	133	5.8	(4.8–6.7)	271	4.6	(4.1–5.1)	
Widow	673	18.7	(17.4–20.0)	401	17.4	(15.8–18.9)	1074	18.2	(17.2–19.2)	
Single	541	15	(13.9–16.2)	407	17.6	(16.1–19.2)	948	16.1	(15.1–17.0)	
Total	3595	100	(–)	2306	100	(–)	5901	100	(–)	
*CD4 category*										< .001
Missing	846	23.5	(22.1–24.9)	727	31.5	(29.6–33.4)	1573	26.7	(25.5–27.8)	
< 200	1592	44.3	(42.7–45.9)	900	39	(37.0–41.0)	2492	42.2	(41.0–43.5)	
200–349	984	27.4	(25.9–28.8)	620	26.9	(25.1–28.7)	1604	27.2	(26.0–28.3)	
350–500	78	2.2	(1.7–2.6)	33	1.4	(0.9–1.9)	111	1.9	(1.5–2.2)	
≥ 500	95	2.6	(2.1–3.2)	26	1.1	(0.7–1.6)	121	2.1	(1.7–2.4)	
Total	3595	100	(–)	2306	100	(–)	5901	100	(–)	
*WHO stage*										0.105
Missing	193	5.4	(4.6–6.1)	105	4.6	(3.7–5.4)	298	5	(4.5–5.6)	
WHO I	707	19.7	(18.4–21.0)	487	21.1	(19.5–22.8)	1194	20.2	(19.2–21.3)	
WHO II	1056	29.4	(27.9–30.9)	645	28	(26.1–29.8)	1701	28.8	(27.7–30.0)	
WHO III	1550	43.1	(41.5–44.7)	993	43.1	(41.0–45.1)	2543	43.1	(41.8–44.4)	
WHO IV	89	2.5	(2.0–3.0)	76	3.3	(2.6–4.0)	165	2.8	(2.4–3.2)	
Total	3595	100	(–)	2306	100	(–)	5901	100	(–)	
*First line regimen*										< .001
Nevirapine	3084	85.8	(84.6–86.9)	1803	78.2	(76.5–79.9)	4887	82.8	(81.9–83.8)	
Efavirenz	489	13.6	(12.5–14.7)	465	20.2	(18.5–21.8)	954	16.2	(15.2–17.1)	
Other	22	0.6	(0.4–0.9)	38	1.6	(1.1–2.2)	60	1	(0.8–1.3)	
Total	3595	100	(–)	2306	100	(–)	5901	100	(–)	
*Art adherence*										0.012
Missing	3112	86.6	(85.4–87.7)	1934	83.9	(82.4–85.4)	5046	85.5	(84.6–86.4)	
Satisfactory	480	13.4	(12.2–14.5)	368	16	(14.5–17.5)	848	14.4	(13.5–15.3)	
Unsatisfactory	3	0.1	(0.0–0.2)	4	0.2	(0.0–0.3)	7	0.1	(0.0–0.2)	
Total	3595	100	(–)	2306	100	(–)	5901	100	(–)	

### Patients lost to follow-up

Among eligible ART patients, 40.6% (n = 2396/5901) were LTFU at any time during the study period i.e. those that missed the last scheduled clinic appointments for at least 90 days and were traced and referred back to the clinic plus those that were never traced. ART patients in the EHR-only group had a LTFU rate of 45.6% (95% Confidence Interval—CI 44.0–47.2) 95% compared to 32.8% (95% CI 30.9–34.7) in the EHR with CDSS group (Table 1).

CDSS was associated with lower LTFU among ART patients (Odds Ratio—OR 0.70 (95% CI 0.65–0.76)). The association was confirmed after adjusting for age-group, sex, marital status, CD4 category and WHO clinical stage, (adjusted Odds Ratio—aOR 0.70 (95% CI 0.65–0.77)). (Table 2). *ART adherence* variable was dropped from the regression model as > 35% of the values were missing.

**Table 2:** Factors associated with lost to follow-up over time among ART patients, N = 5901

Characteristic	Unadjusted odds ratios			Adjusted odds ratios		
	OR (95% CI)	P value	Global p value	OR (95% CI)	P value	Global p value
*Site status*						
Control intervention	0.70 (0.65–0.76)	< .001	< .001	0.70 (0.65–0.77)	< .001	< .001
*Age group, years*						
< 10 years						
10–19 years	1.41 (1.13–1.77)	0.003	0.127	1.2 (0.95–1.5)	0.126	0.187
20–29 years	0.88 (0.76–1.02)	0.087		0.88 (0.75–1.04)	0.129	
30–39 years	0.94 (0.82–1.08)	0.393		0.94 (0.8–1.12)	0.499	
40–49 years	1.14 (0.98–1.33)	0.093		1.01 (0.85–1.21)	0.872	
50–59 years	0.92 (0.77–1.1)	0.376		0.9 (0.74–1.11)	0.335	
60 + years	1.12 (0.86–1.46)	0.4		1.11 (0.83–1.47)	0.482	
*Sex*						
Male						
Female	1.01 (0.94–1.09)	0.713	0.713	0.95 (0.87–1.03)	0.193	0.193
*Marital status*						
Divorced/Separated						
Married	1.55 (1.25–1.92)	< .001	< .001	1.34 (1.11–1.61)	0.002	< .001
Single	2.97 (2.37–3.72)	< .001		1.39 (1.13–1.72)	0.002	
Widow	1.56 (1.25–1.94)	< .001		1.43 (1.17–1.74)	0.001	
*CD4 category*						
< 200						
200–350	1.49 (1.36–1.65)	< .001	0.678			
350–500	1.07 (0.82–1.39)	0.633				
≥ 500	0.98 (0.75–1.28)	0.853				
*WHO stage*						
WHO IV						
WHO I	2.75 (2.13–3.55)	< .001	< .001	1.24 (0.98–1.58)	0.076	0.026
WHO II	1.71 (1.33–2.2)	< .001		1.36 (1.08–1.72)	0.01	
WHO III	1.35 (1.05–1.73)	0.019		1.27 (1.01–1.6)	0.044	
*First line regimen*						
Other						
Efavirenz	1.90 (1.09–3.32)	0.024	< .001	1.83 (1.04–3.21)	0.036	< .001
Nevirapine	3.18 (1.84–5.51)	< .001		2.82 (1.62–4.91)	< .001	

### Proportion of patients linked back to treatment

The overall proportion of ART patients that were LTFU and were traced and linked back to treatment was 25.8% (95% CI 24.2–27.3). The proportion of ART patients linked back to treatment were 23.3% (95% CI 21.5–25.0) and 30.6% (95% CI 27.9–33.4) in the EHR-only and EHR with CDSS sites respectively. CDSS was associated with a higher likelihood of return to treatment among those LTFU (OR 1.46 (95% CI 1.24–1.72)). After adjusting for Age, Sex, Marital status, WHO clinical stage and Regimen, we showed a statistically significant positive association between CDSS and linkage to treatment among patients that were LTFU (aOR 1.53 (95% CI 1.28–1.82))—*p* < 0.001.

### Time from study enrollment to documentation of LTFU

**Fig. 2:**
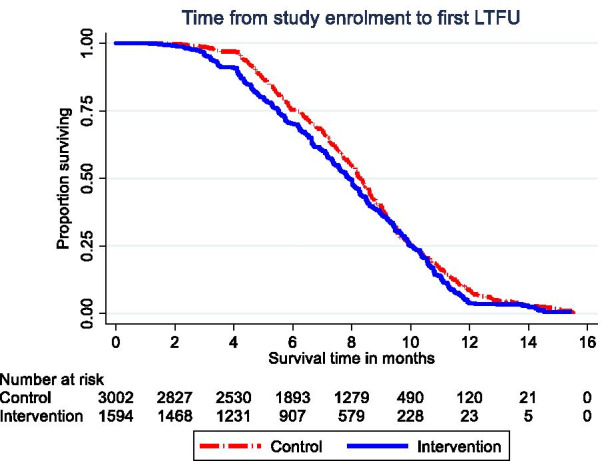
Time from study enrollment to documentation of first LTFU

The respective median times from study enrollment to documentation of first LTFU in the EHR-only and EHR with CDSS sites were 8.28 months (IQR—IQR: 6.14–10.05) and 7.92 months (IQR 5.52–9.98). Figure 2 shows the time from ART initiation to the first LTFU. The results from the Cox regression models (not included in tables) were presented as hazard ratios associating a CDSS to time from study enrollment to documentation of first LTFU: (hazard ratio—HR 0.87 (95% CI 0.81–0.93)). After adjusting for age-group, sex, marital status, CD4 category and WHO clinical staging, CDSS was associated with a reduction in time from enrollment to documentation of first LTFU (aHR 0.85 (95% CI 0.78–0.92)).

## Discussion

Our study showed that clinics with a CDSS had a 30% lower proportion of ART patients who were LTFU compared to those without a CDSS. Nearly half of the patients actively receiving ART had been lost to follow up at least once during the 12 month study period. This is similar to study by Clouse et al.[20]. The CDSS generated alerts that were printed out and placed in the individual patient charts to notify the clinical staff when patients missed their appointments (classified by the Kenyan Ministry of Health guidelines as defaulters) [11] and appropriate recommendations that were provided to the clinical staff and social workers. Based on the finding of our study we believe that the individual patient level alerts may have been effective in early identification of defaulting patients leading to immediate tracing action and getting them back to the clinic before they could be classified as LTFU.

The proportion of ART patients that were LTFU at least once and were traced and linked back to treatment was higher in the sites with a CDSS than those with EHR only. Printouts/alerts in the individual patient charts generated by the CDSS reminded the clinical staff to intensify efforts, in collaboration with peer educators, community health and social workers to trace the LTFU patients, counsel and link them back to treatment. This is consistent with studies by Wilson et al.and Semeere et al., which showed that use of electronic health records was associated with effective tracing of patients that are LTFU [21, 22]. The CDSS was marginally associated with reduced time from study enrollment to first documented LTFU. The duration that a patient takes before they are LTFU is influenced by several factors; other studies show that patient’s behavioral characteristics, clinical processes, and accessible and client-friendly services could contribute to patients becoming defaulters or LTFU [9, 23]. Early documentation of LTFU through EMR-generated alerts which were printed out and inserted in individual patient charts prompted earlier follow-up by the peer educators and community health workers.

HIV patients who are lost to follow-up are unlikely to adhere to treatment guidelines and to achieve viral suppression. This comes with potential risks like higher mortality due to treatment failure, co-infection with opportunistic illnesses and likelihood of transmission of HIV to uninfected sexual partners [24]. Interventions such as use of enhanced vital statistics and patient encounter simulations have been shown to enhance patient retention and improve linkage of chronic care patients who are LTFU back to treatment in resource-limited settings [21, 22]. Integration of innovative solutions such as short message system (SMS) based reminders sent directly from an EHR’s CDSS to a patient’s cell phone improves clinic attendance, compliance with medication and other positive behavior which lead to better treatment outcomes [25]. Our study, based on a stronger design and large sample size, provides early evidence of the important role that individual patient level alerts through a CDSS plays in improving adherence to HIV treatment guidelines and reducing LTFU in resource-limited settings. For a country like Kenya which had about 1 million patients receiving ART at the end of 2018, the use of a CDSS has the potential to reduce the LTFU of up to 128,000 patients (40% reduction of LTFU) and contribute to the tracing and linking back to care up to nearly 40,000 patients that are LTFU (approximately 33% of all patients LTFU). As the number of HIV patients receiving ART increases following the release of the 2016 Edition of WHO’s consolidated guidelines on the use of antiretroviral drugs for treating and preventing HIV infection that recommend universal treatment for all HIV infected persons irrespective of age, clinical presentation or pregnancy status [18] the use of a CDSS to enhance quality of care and reduce LTFU becomes indispensable. This need is more acute in sub-Saharan Africa where HIV disease burden is highest and health systems to support treatment programs weakest.

Our study had a few limitations: As the study was conducted in Ministry of Health-owned health facilities which often have limited resources such as staffing, reliable electric power and key supplies, documenting all clinic attendance and results of patient tracking was often challenging. These challenges affected the study sites in equal measure. During the data collection period, the KEMRI staff directly involved in the study ensured that missing data were collected during subsequent patient visit and recorded as accurately as possible. Nearly a third of the patient records did not have critical follow-up data and were excluded from analysis as we could not ascertain whether they were for those LTFU after the first visit, transit patients or those who had not yet decided to permanently enroll for HIV care at the health facilities in the study. The sample size for this study was quite large, enhancing the precision of results generated from the analyses. Our study showed that only one-third of the patients that were LTFU were linked back to HIV care or treatment. Although this figure is low, sites with a CDSS implementation had a much higher proportion of patients traced and linked to care and treatment compared to those without. It is worth noting that a CDSS is only one of the potential solutions for reducing LTFU and tracing of patients and should be implemented together with other interventions such as enhanced patient education, provider related characteristics (e.g. improved patient waiting time, streamlined services) and client-friendly services that help improve clinic attendance as recommended in several studies [9, 23, 25].

Since 2014, Kenya has implemented interventions such as improved contract tracing through patients’ mobile phones, enhanced adherence counseling, innovative community support services to improve retention and reduce LTFU. In 2018, the country introduced multi-month prescription and dispensing of drugs among stable patients with the aim of reducing the number of clinic visits and decongesting clinics thereby reducing waiting time. Future studies should investigate which co-interventions work in low resource settings.

## Conclusion

An Alert-based CDSS implemented as part of an EHR can contribute to enhanced quality of HIV treatment through reduction and early documentation of defaulting and LTFU among HIV patients receiving ART, so that follow-up to re-engage clients in care can be activated in resource-limited settings in Kenya. Future research is needed on how CDSS can best be combined with other interventions to reduce LTFU.

## Data Availability

The datasets generated and/or analyzed during this study are not publicly available in line with the KEMRI Guidelines. However, the corresponding author may seek KEMRI’s permission to share the de-identified data upon reasonable request.

## Abbreviations

ANOVA: Analysis of variance
ART: Antiretroviral therapy
CCA: Complete case analysis
CDC: US Centers for Disease Control and Prevention
CDSS: Clinical decision support systems
CD4: Cluster of differentiation 4
CI: Confidence interval
C-PAD: Comprehensive Care Centre Patient Application Database
EHR: Electronic health records
HR: Hazard ratio
aHR: Adjusted hazard ratio
IQR: Inter-quartile range
KEMRI: Kenya Medical Research Institute
LTFU: Lost to follow-up
MCAR: Missing completely at random
MOH: (Kenyan) Ministry of Health
OR: Odds ratio
aOR: Adjusted odds ratio
SSA: Sub-Saharan Africa
UNAIDS: United Nations Joint Committee on AIDS
WHO: World Health Organizations
